# The Emerging Role of Ionic Liquid-Based Approaches for Enhanced Skin Permeation of Bioactive Molecules: A Snapshot of the Past Couple of Years

**DOI:** 10.3390/ijms222111991

**Published:** 2021-11-05

**Authors:** Ana Gomes, Luísa Aguiar, Ricardo Ferraz, Cátia Teixeira, Paula Gomes

**Affiliations:** 1 LAQV-REQUIMTE, Departamento de Química e Bioquímica, Faculdade de Ciências, Universidade do Porto, Rua do Campo Alegre 687, P-4169-007 Porto, Portugal; anagomes@fc.up.pt (A.G.); luisa.silva@fc.up.pt (L.A.); ricardoferraz@eu.ipp.pt (R.F.); catia.teixeira@fc.up.pt (C.T.); 2 Ciências Químicas e das Biomoléculas, CISA, Escola Superior de Saúde, Politécnico do Porto, R. Dr. António Bernardino de Almeida 400, P-4200-072 Porto, Portugal

**Keywords:** active pharmaceutical ingredients, complicated skin and soft tissue infections, cosmetics, ionic liquids, peptides, transdermal delivery, wound-healing

## Abstract

Topical and transdermal delivery systems are of undeniable significance and ubiquity in healthcare, to facilitate the delivery of active pharmaceutical ingredients, respectively, onto or across the skin to enter systemic circulation. From ancient ointments and potions to modern micro/nanotechnological devices, a variety of approaches has been explored over the ages to improve the skin permeation of diverse medicines and cosmetics. Amongst the latest investigational dermal permeation enhancers, ionic liquids have been gaining momentum, and recent years have been prolific in this regard. As such, this review offers an outline of current methods for enhancing percutaneous permeation, highlighting selected reports where ionic liquid-based approaches have been investigated for this purpose. Future perspectives on use of ionic liquids for topical delivery of bioactive peptides are also presented.

## 1. Introduction

**Figure 1:**
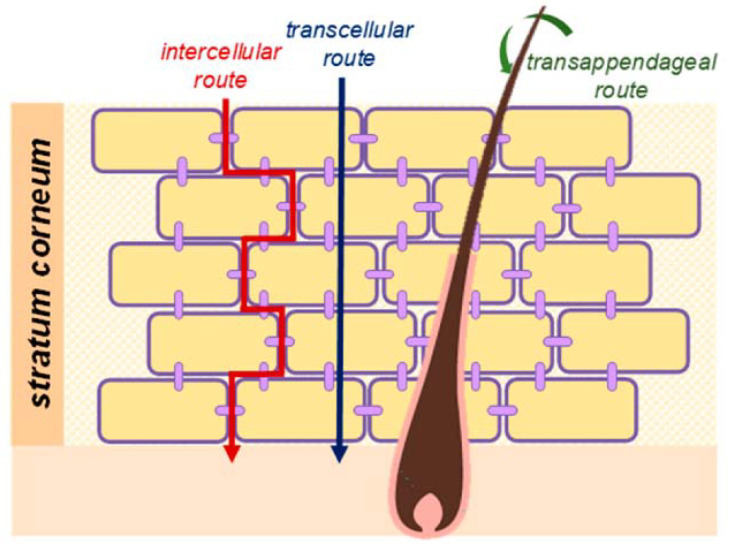
Schematic view of skin permeation pathways: intercellular, transcellular, and transappendageal [4]. Reprinted with permission from ref. [4]. Copyright 2021 MDPI.

The skin is the largest organ of the human body, protecting it against external aggressions while keeping its thermal regulation and conveying the sense of touch. Being such a formidable barrier, the skin may also be a considerable obstacle for efficient topical absorption and/or transdermal delivery of many active pharmaceutical ingredients (APIs) and cosmetics. Different strategies have been developed and explored over time to promote dermal permeation of different substances, since empirical formulations developed over five thousand years ago in the ancient Egyptian and Babylonian civilizations, to cutting-edge physical, chemical, and [bio]nanotechnological approaches that build on current knowledge of skin physiology, composition, and permeation routes (Figure 1) [1,2,3].

**Figure 2:**
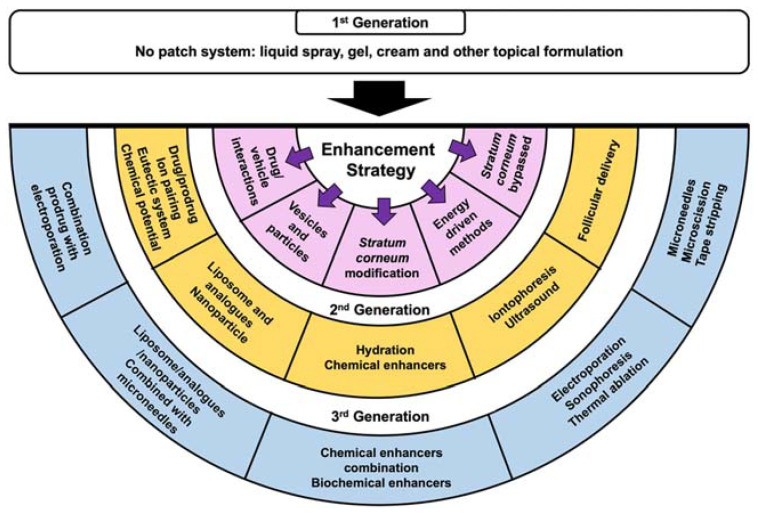
Diagram on the progress in the development of percutaneous absorption enhancement strategies [5]. Reprinted with permission from ref. [5]. Copyright 2021 Springer Nature.

The evolution of strategies to promote percutaneous penetration of molecules and macromolecules is schematized in Figure 2 [5]. Current approaches encompass: (i) physical methods like sonophoresis, iontophoresis, thermophoresis, electroporation, laser microporation, thermal ablation, or microneedle patches [6]; (ii) encapsulation in suitable nanocarriers (nanoparticles, liposomes, ethosomes, niosomes, aquasomes, etc.) [7]; (iii) use of engineered controlled-release and/or stimuli-responsive materials (patches, wearable devices, and others) [8]; and (iv) addition of [bio]chemical permeation enhancers (e.g., fatty acids, fatty alcohols, alcohols, glycols, peptides, [bio]surfactants) [9]. Some of these methods can be combined, to further improve the percutaneous permeation efficiency.

Chemical permeation enhancers (CPEs) act through interactions with molecules that compose the stratum corneum (SC), the outermost layer of the skin that limits the rate of dermal/transdermal permeation. CPEs include different chemical families, such as alcohols (e.g., isopropyl alcohol), glycols (e.g., propylene glycol), terpenes and terpenoids (e.g., menthol), essential oils (e.g., eucalyptus), sulfoxides (e.g., dimethylsulfoxide), ether alcohols (Transcutol^®^), and amides (Azone^®^), among many others [9]. The over 600 CPEs reported to date act via different pathways, the most common of which is disturbance of the cell membrane phospholipid bilayers [10]. As such, it comes with no surprise that an important portion of CPEs regard amphiphilic molecules such as fatty acids and respective esters, fatty alcohols, and several other anionic, cationic, zwitterionic, and non-ionic surfactants (Table 1) [9].

Cetylpyridinium chloride (Table 1) is one of the amphiphilic CPEs most commonly used in cosmetics, where it also acts as a preservative and antiseptic, due to its antimicrobial properties [11]. Cetylpyridinium chloride, by being an ionic pair that combines an organic cation (cetylpyridinium) with an inorganic (could be organic) anion, has the structural type of an ionic liquid. Ionic liquids (ILs) are generally defined as organic salts composed by an organic cation and an organic or inorganic anion that are stable below their melting point. ILs are known by their remarkable physical and chemical properties and by their possible customization, since they can be designed to exert the desired effect by the correct choice of the ions that compose them [12].

The relevance of ILs is currently consolidated in many areas that explore them as (i) greener alternatives to common organic solvents, (ii) task-specific materials, and (iii) polyvalent players in pharmaceutical sciences. In the latter field, a wide range of ILs have been developed, spanning from ILs with intrinsic bioactivity to those having suitable properties for drug formulation and transport [13,14]. In this regard, ILs have been advanced as high value mediators of dermal and transdermal delivery (DTD) of small and large molecules, whose properties can be tailored through a few simple design principles as recently proposed by Mitragotri and co-workers [15].

In view of the above, this review offers a snapshot of the latest developments regarding the most common methods for DTD of [bio]pharmaceuticals, highlighting the emerging role of ILs for enhancement of percutaneous absorption of diverse payloads, including proteins and peptides.

## 2. Overview of Current Methods for Dermal and Transdermal Drug Delivery

### 2.1. Physical Methods

**Figure 3:**
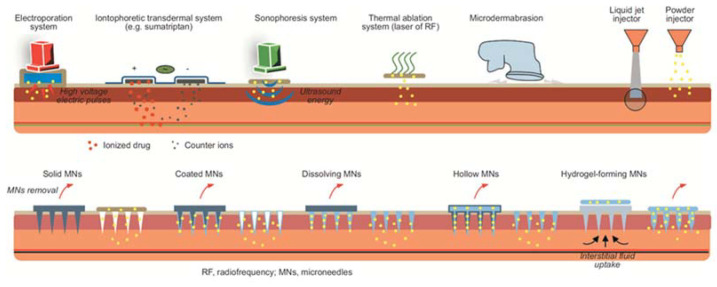
Physical methods for enhancement of percutaneous absorption [1]. Reprinted with permission from ref. [1]. Copyright 2019 Bentham Science.

Physical methods to enhance percutaneous permeation can be divided into indirect and direct approaches (Figure 3). In indirect methods, different types of energy are used to promote penetration and diffusion of bioactive solutes through the SC. Thus, electrical energy is applied in electroporation and iontophoresis procedures, acoustic energy is used in sonophoretic methods (e.g., cavitation, ultrasound pressure), and laser or magnetic energies can also be applied in this context. 

**Electroporation** uses high-voltage electrical pulses to generate transient pores in cell membranes through which a wide variety of substances, from small drugs to nucleic acids, can reach the intracellular milieu. Latest examples on skin applications of electroporation techniques include the permeation of low concentration formic and acetic acids for wound disinfection [16,17]. This type of electroporation applications was found to promote differential regrowth of dermal fibroblasts and keratinocytes [18].

**Iontophoresis** is based on use of mild electrical currents to increase skin permeation, mostly by electromigration of ions within the electric field applied, but also by electroosmosis or, to a minor extent, to enhanced passive diffusion. Understandably, this method is better suited for the permeation of charged molecules, whose transport can be modulated by a number of parameters [19]. Ongoing investigational work on biomedical applications of iontophoresis spans from pre-clinical studies on transdermal delivery of anti-hypertensive agents [20], to clinical trials on iontophoresis of treprostinil as a potential treatment for diabetic foot ulcers (DFUs) [21].

**Sonophoresis** has been also thoroughly explored for enhancement of percutaneous absorption, mainly—but not exclusively—through cavitation or ultrasound (US)-based techniques. Both cavitation [22] and US-based [23] sonophoretic methods have been also recently considered for wound healing approaches, with encouraging results, including in diabetic mice [23]. Of note, care must be taken in order to avoid that the intensity and duration of US irradiation are high enough to cause burns at the irradiation site [1].

In direct methods, though considered a little invasive, pores are created in the SC, through which the entry of solutes into the epidermis and eventually the dermis is forced by means of mechanical, thermal, or pressure-based approaches. Currently, the most popular direct methods include (i) the use of different types of microneedles (MNs)—solid, coated, hollow, dissolving, or hydrogel-forming—to force percutaneous permeation of bioactive compounds, and (ii) microdermabrasion (mechanics-driven) and thermal ablation (heat-driven). Other physical methods have been used to promote percutaneous permeation, though to a lesser extent; one example is that of jet injectors, whereby solid, liquid, or plasma jets force drug delivery by means of the high pressure exerted when they hit the skin [1].

**Microneedles** (MNs)-based approaches are amongst the most widely explored physical methods to promote DTD. In recent years, different types of MN arrays have been thoroughly studied as hurtless alternatives to classical injections, being well advanced in the clinics [1,24]. MNs-based technologies are being pushed forward mainly for transdermal delivery of peptides, proteins, and antibodies [25], and for cosmetic applications [26]. Still, the latest reports highlight the therapeutic potential of MNs to tackle non-healing wounds [27]. Based on the promising performance of diverse MN arrays such as Manuka honey, MNs are able to promote healing and exert potent bactericidal action against methicillin-resistant *S. aureus* (MRSA) [28]. 

**[Photo]thermal ablation** techniques are also regarded as a promising way to enhance percutaneous permeation of therapeutics. In classical thermal ablation approaches, components of the outermost skin layer are literally vaporized upon ultrafast exposure to an extremely high (>300 °C) temperature, which disrupts the SC barrier, facilitating subsequent absorption of the drug, typically administered through a transdermal patch. [Photo]thermal ablation methods have been also considered for tackling mild to severe skin infections [29], by means of (i) direct ablation of the microbial pathogens [30], (ii) sensitization of bacterial biofilms to standard antibiotics [31], or (iii) accelerating wound healing [32,33]. One recent example advances a laser-activatable nanosystem able to exert a potent action against multidrug resistant (MDR) bacteria often associated to non-healing wounds, by a combined photothermal effect and controlled release of copper(II) ions [34].

The distinct physical methods mentioned above act through different permeation mechanisms and have been employed alone or combined, with each other or with non-physical methods, to enhance the absorption of a wide range of APIs, and also cosmeceuticals, through the skin [1,9,35,36].

### 2.2. Non-Physical Methods—Nanosized Delivery Systems

**Figure 4:**
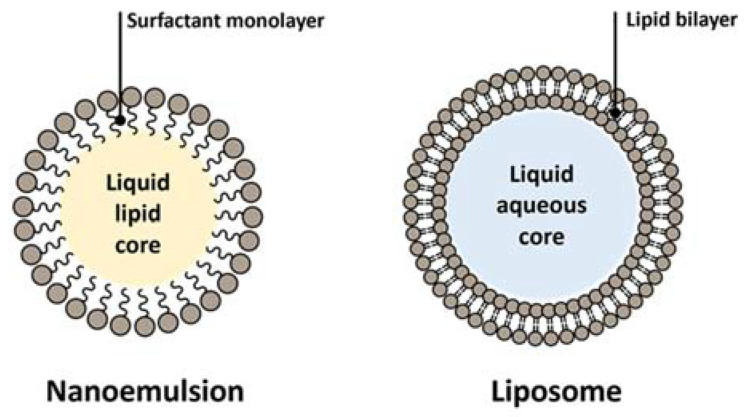
General representation of nanoemulsion and liposome.

Nanosized [bio]materials and formulations are regarded as the gold standard of non-physical methods for drug delivery, with emphasis on dermal and transdermal applications. Lipid-based or -inspired nanosystems are by far the most common, covering from the classical examples of nanoemulsions and liposomes (Figure 4) to solid lipid nanoparticles and nanostructured lipid carriers. 

**Figure 5:**
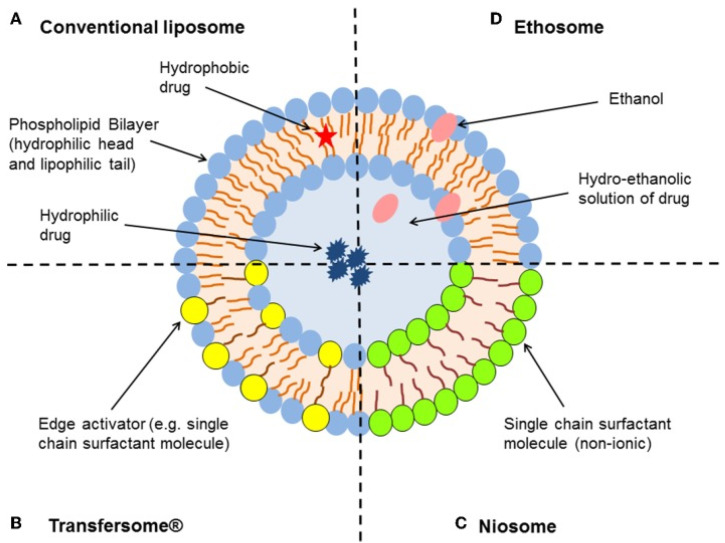
Examples of liposome-inspired vesicles [45]. Reprinted with permission from ref. [45]. Copyright 2015 Hua.

**Nanoemulsions** (NEs) allow the dispersion of drug-containing droplets in very high interfacial areas, and their relevance towards the enhancement of percutaneous absorption of either hydrophilic (water-in-oil NEs) or lipophilic (oil-in-water NEs) substances is well established [37,38]. NEs in use or under investigation today offer diverse compositions and degrees of complexity, based on diverse amphiphiles, from natural lipids to synthetic surfactants, and combinations thereof [37,38]. Use of essential oils in the formulation of NEs has also been addressed. For instance, a clove oil-based NE was developed to combine the anti-inflammatory and antifungal properties of the main oil component, eugenol, with those of the drug cargo, naftifine, used to tackle skin fungal infections [39]. Lately, other types of liposome-inspired vesicles (Figure 5), such as ethosomes, transfersomes, niosomes, among other, have been advanced [1,3,40,41]. Lipid-based/inspired nanosystems stem from ancient knowledge on the value of fatty acids and derived hydrolyzable lipids, especially phospholipids, to enhance percutaneous absorption of a variety of substances for both health- and beauty-care. Phospholipid-based emulsions, as well as micellar and liposomal formulations for skin care are all around us, hence it comes as no surprise that lipid-based/inspired nanocarriers have a prominent role in current approaches for DTD of [bio]pharmaceuticals [1,3,40,41,42,43]. Still, nanosystems that do not [exclusively] derive from natural lipids have been thoroughly explored for percutaneous permeation of bioactive molecules; organic nanoformulations comprising either natural or synthetic polymers and/or surfactants other than natural lipids can be found in recent literature, covering distinct types of dendrimers, nanoparticles (NPs), nanoemulsions (NEs), micelles, and hydrogels, for diverse purposes. Likewise, inorganic nanosystems, including metallic, non-metallic, and magnetic NPs, among others, have been employed for DTD of [bio]pharmaceuticals [42,44].

**Vesicular nanocarriers** (VNs) currently encompass a myriad of different, mostly spherical, structures that share the common trait of having single or multiple bilayer lamellae separating one or more aqueous or hydroalcoholic inner compartments from the outer medium, and which have been widely used for topical disorders and cosmetics [9,46]. The best known and most commonly employed VNs are liposomes, spherical colloidal bilayer structures formed spontaneously by amphipathic phospholipids in aqueous environments, which were originally reported in 1965 [47]. However, a variety of liposome-inspired VNs has evolved since, such as:

- *ethosomes*, soft VNs whose structure is closely related to that of liposomes, the main difference being the presence of ethanol in moderate to high concentrations, which confers the vesicles high malleability and the ability to significantly enhance the percutaneous permeation of highly lipophilic molecules [48];
- *transfersomes*, ultraflexible VNs composed by either phospholipids or other bilayer-forming amphipathic molecules packed together with edge activators that decrease the vesicle’s interfacial tension; this conveys a very high elasticity enabling a much better permeation through the SC probably via the intercellular route [49];
- *niosomes* are VNs usually composed by cholesterol and alkyl or polyglycerol-based non-ionic surfactants that usually offer higher osmotic stability and involve lower production costs as compared to phospholipid-based VNs. Yet, like in liposomes, the physical stability of niosomes is not adequate for prolonged storage [50,51].
- *other “somes”* are continuously emerging as novel VNs for drug delivery, particularly for topical applications. Among others, these comprise: invasomes—terpene-containing ethosome analogues with enhanced penetration into deeper layers of the skin [52]; aspasomes—multilayered VNs formed by combination of ascorbyl palmitate with cholesterol and charged lipids [53]; and other self-assembling hollow nanostructures based on, e.g., solid crystalline cores (aquasomes), liquid crystalline phases (cubosomes, hexosomes), or hollow coagulated nanoparticles (colloidosomes) [54], as well as polymer-based vesicles (polymersomes) [55].

All these types of artificially produced VNs have been recently explored and optimized to create increasingly efficient formulations for DTD of bioactive molecules and macromolecules. For instance, different hydrogels have been investigated as suitable vehicles for topical delivery of drug-loaded niosomes [56]. In another recent report, an ethosome-based gel showed good performance both in vitro and in vivo for delivery of thymosin β-4, a protein that is relevant for wound repair [57]. Less common VNs have been lately advanced for diverse purposes, such as unsaturated fatty-acid based nanovesicles (ufasomes) for DTD of terbinafine hydrochloride to address *Candida albicans*-associated topical fungal infections [58]. Interestingly, when looking at the forefront of VNs-mediated drug delivery, we can witness the increasing relevance of cell-based VNs such as exosomes [59] or outer membrane vesicles (OMVs) from Gram-negative bacteria [60], whose major hallmark is their expectedly high biocompatibility and low immunogenicity. Exosomes are extracellular vesicles that integrate proteins and nucleic acids of their secreting cells, and which can affect function and properties of other cells able to internalize them [59]; as such, recent efforts address their engineering for targeted drug delivery [61,62], also for diverse dermatological applications [63,64,65] and wound healing [66]. Besides exosomes, OMVs are also becoming prominent actors in several biomedical applications, including recent examples where engineered OMVs from transgenic *Escherichia coli* have been used for DTD of biopharmaceuticals, alone [60] or in combination with phototherapy [67].

**Figure 6:**
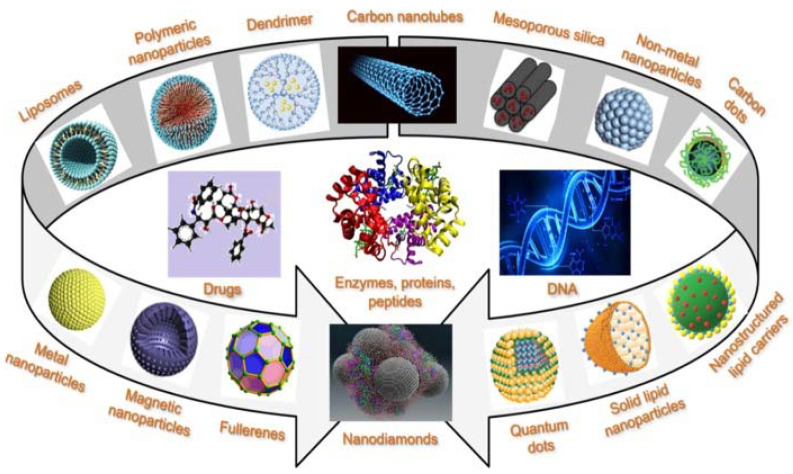
Different types of particulate systems with potential interest as nanocarriers for targeted delivery of drugs and biological macromolecules [68]. Reprinted with permission from ref. [4]. Copyright 2019 American Chemical Society.

**Nanocarriers** (NCs) include many other structures besides bilayered VNs, with diverse properties and applications, including targeted delivery of [bio]pharmaceutics (Figure 6) [68]. NCs are typically colloidal particulate systems having at least one dimension not larger than 100 nm. NCs can be categorized in different ways, i.e., based on morphology (nanorods, nanoshells, nanocages, nanostars, etc.), core material (metallic, ceramic, polymeric, carbon-based, lipid-based, etc.), specific physical and/or chemical properties (magnetic, [semi-]conductivity, thermoconductivity, stiffness, porosity, etc.), among other criteria (e.g., surface charge or functionalization) [69]. It should be outlined that most current nanodelivery approaches rely on hybrid/composite systems often comprising inorganic, biopolymeric, and several other natural and/or synthetic organic components, which blur the frontiers between different types of NCs. While nanosized liposomal and liposome-like VNs addressed in the previous section are also NCs, this section is aimed at pinpointing a few recent cases where NCs with non-bilayer structures have been explored for topical delivery focusing on the treatment of skin and soft tissue infections and wound-healing enhancement.

**Figure 7:**
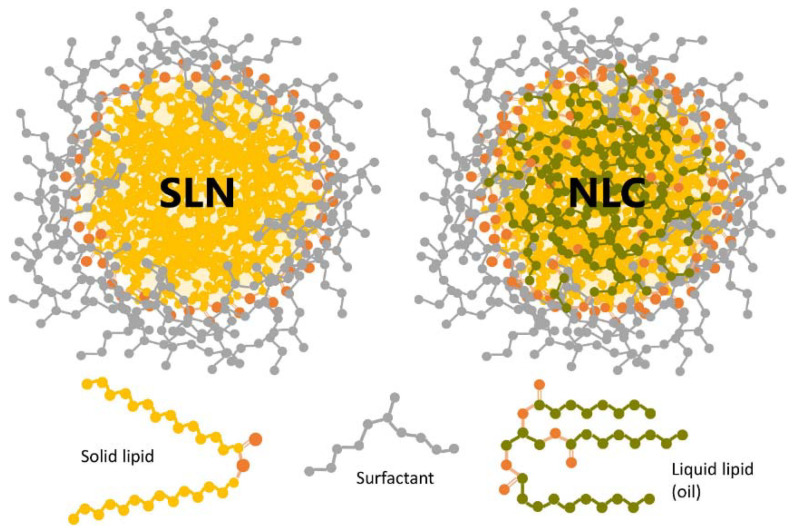
General structures of SLNs and NLCs [72]. Reprinted with permission from ref. [72]. Copyright 2020 Scioli Montoto, Muraca and Ruiz.

Lipid-based NCs, of which solid lipid nanoparticles (SLNs) and nanostructured lipid carriers (NLCs) are the most representative examples (Figure 7), are currently regarded as enhanced alternatives to classical liposomal-based formulations. SLNs emerged as the first-generation of non-liposomal lipid-based nanocarriers, featured by a spherical shape and a payload-containing solid core stabilized by an outer layer lined with surfactants. SLNs offer many advantages over other types of NCs, the main of which are their high biocompatibility and biodegradability, but also high compatibility with a wide range of payloads and cost-effective production, among others. Still, the solid nature of SLNs poses a few limitations, such as low loading capacity and the risks of premature drug release, unexpected gelation, and crystallization upon storage. NLCs emerged to circumvent these hurdles, by offering a nanostructured core containing low-melting point lipids (liquid at room temperature) [70,71,72].

The potential of both SLNs and NLCs for drug delivery, including across the formidable obstacles that are the blood–brain barrier and the skin, has been widely addressed [71,72,73]. Promising results have been lately reported to DTD of anti-inflammatory, antibacterial, antiviral, antifungal, antiacneic, and anticancer agents, among others, as nicely reviewed by Souto et al. [73]. Also, SLNs and NLCs have been explored for DTD of cosmetics, with encouraging performances [74]. Importantly, the formulation of SLNs and NLCs for DTD of [bio]pharmaceuticals and cosmeceuticals can be easily tailored according to their specific application and payload [73,74].

SLNs and NLCs are also showing promise to promote skin regeneration [75,76,77]. Recent investigations focused on DTD of essential oils having healing effects due to either a dual anti-inflammatory/collagenesis-inducing action of the oil itself [78], or to a synergic antimicrobial and healing action elicited by combination of the essential oil with specific components in the lipid nanocarrier (e.g., oleic acid) [79]. Very recently, different SLNs and NLCs have been tested along with NEs for DTD of curcumin, a hydrophobic photosensitive phytomolecule, whose anti-inflammatory, antimicrobial, and healing properties have been known for long [80]. This study revealed that all tested NCs were efficient for photoprotection of the phytopharmaceutical, with NLCs offering the best pharmacological performance, provided the matrix fluidity was tuned for optimized skin occlusion and drug release rate [80]. In line with this, multiple benefits of SLNs/NLCs-mediated delivery of natural and synthetic antimicrobials have been advocated in the recent literature [81,82,83], including for fighting multidrug-resistant (MDR) infections [84,85]. Thus, a new “nanoantibiotic era” is on the rise [86] that will likely have great impact on the management of complicated skin and skin structure infections (cSSTI), given the unique ability of lipid-based NCs to overcome the skin barrier [87]. One illustrative example is “nanoRIF”, a rifampicin-loaded hybrid lipidic/polymeric NC that showed in vivo efficacy against *Staphylococcus aureus*-associated infection on skin [88].

*Non-lipidic NCs* enclose a broad variety of organic and inorganic nanomaterials, a few of which are next highlighted for their recent interest for the enhancement of percutaneous permeation:

- *dendrimers* are hyperbranched arborescent spherical NPs that may be composed by either natural (e.g., amino acid- or peptide-based) or artificial (e.g., ethylene glycol-based) dendrons, whose individual structure and tridimensional arrangement in the final dendrimer have great impact on the physical, chemical, and drug loading/release properties of the whole nanosystem [89]. The biomedical relevance of dendrimers, including for drug delivery, is growing exponentially [90,91], and interest is now falling on topical applications. Latest reports in this regard concern, e.g., use of poly(amidoamine)- or PAMAM-based dendrimers for enhanced skin permeation of the chlorhexidine digluconate antiseptic [92], or dermal delivery and follicular targeting of the antiacneic agent adapalene [93].
- *[bio]polymer-based NCs,* encompassing [bio]polymeric NPs, films, gels, nanofibers, among others, have been thoroughly investigated for drug delivery applications [94], including for topical use [95]. Natural polymers such as chitosan, poly(glycolic acid), poly(lactic acid), hyaluronic acid, and poly(arginine) are amongst the most popular components of polymeric NCs, given their biocompatibility and biodegradability, along with their chemical “flexibility” to enable the production of a wide range of multi-component customized stimuli-responsive nanomaterials [95]. Chitosan-based polymeric NCs have been thoroughly explored for topical applications, given the intrinsic antimicrobial and healing properties of chitosan [96]. One recent example concerns development of a chitosan/carboxymethyl cellulose-based nanogel for transdermal co-delivery of atorvastatin and *Nigella sativa* oil for wound management, taking advantage of the anti-inflammatory, immunomodulatory, antioxidant, and antibacterial properties of both bioactive cargoes; in vitro permeation, cytotoxicity, healing, and bactericidal activity assays delivered quite promising results [97]. Many other polymer-based nanomaterials have been explored in recent years for topical applications from, e.g., extracellular matrix-mimicking nanofibrous scaffolds [98] to promote accelerated healing of chronic wounds.
- *inorganic NCs* integrate metal-, metal oxide-, and mesoporous silica-based NPs as the most popular examples, although other nanosystems, such as inorganic polymer-based NCs, carbon-, carbon oxide-, or black phosphorus-based nanomaterials, also fit this category [99,100,101,102]. Although the majority of inorganic NCs in drug delivery, including DTD, has been addressed to cancer theranostics [103] they have also been explored for antimicrobial therapies [104], where silver-based NPs (AgNPs) occupy a prominent role, given the intrinsic antibacterial properties of silver [52,105]. For instance, AgNPs synthesized from silver nitrate in the aqueous extract of a medicinal plant (*Acanthospermum australe*) used in South America to treat cSSTI, were recently reported to have potent wide spectrum antimicrobial activity [106]. Yet, AgNPs, as well as other inorganic NPs such as zinc oxide-based ones, may pose toxicity issues for dermatological and dermocosmetic use [107]. As such, recent reports on use of AgNPs, or even of other inorganic NCs, for DTD of [bio]pharmaceuticals are relatively scarce. Notwithstanding, graphene oxide-based NCs have been lately highlighted for topical applications [108], including as bioactive agents able to tackle cSSTI per se [109,110]. Other carbon-based inorganic NCs have also been explored to tackle skin disorders, with emphasis on wound healing and control of cSSTI, as recently revised elsewhere [111]. Additional examples on use of inorganic NCs for DTD of [bio]pharmaceuticals mainly address combination with physical methods, in particular with microneedle-based technologies [112,113,114].

### 2.3. Chemical Permeation Enhancers

**Figure 8:**
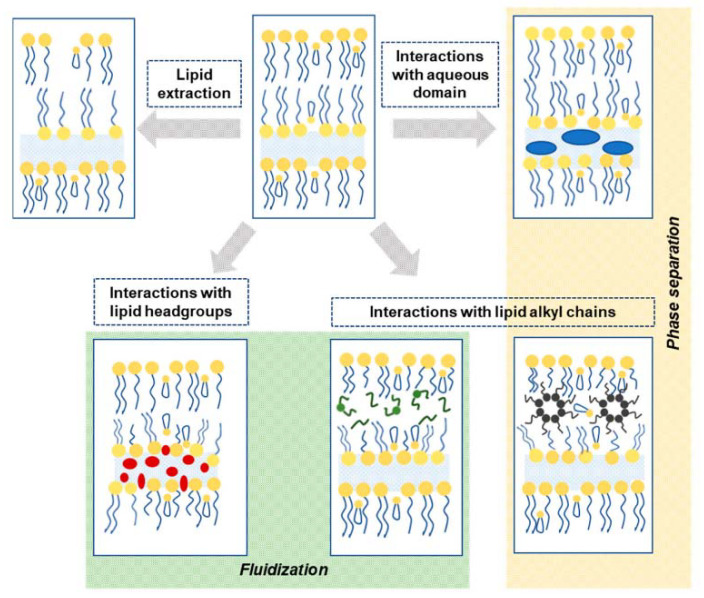
Schematic view of the modes of action of CPEs [4]. Reprinted with permission from ref. [4]. Copyright 2021 MDPI.

As already mentioned, chemical permeation enhancers (CPEs) are molecules able to temporarily alter the structure of the SC, thus enhancing the percutaneous permeation of different substances. The performance of a CPE depends on its ability to both efficiently partition from the applied medium into the skin lipid layer and interact with the constituents of this layer, causing momentary but significant perturbations that lead to the desired higher permeability of the SC. While the solution-to-SC partition is influenced by the lipophilicity of the CPEs, the size and structure of the latter dictate the permeation enhancement pathway(s) (Table 2, Figure 8). Thus, some CPEs, like alcohols and polyols, act mainly as solvents that increase the solubility of the drug and its partitioning into the SC, whereas other solvents used as CPEs, as dimethylsulfoxide, are further able to extract lipid molecules from the SC, creating channels that turn the SC more permeable. Other CPEs, like fatty acids or their esters, are able to insert into the bilayer structures of skin lipids, altering their ordered packing and thus increasing permeability [10,115,116]. Relevantly, though CPEs are usually associated to lipophilic or amphiphilic organic compounds (Table 2), water is the safest and most widely employed CPE for dermatological and dermocosmetic applications; water offers the simplest way to deliver hydrophilic compounds across the skin, but is also able to enhance the permeation of lipophilic ones, as it can both interact with the polar head groups in the SC lipid bilayer and disrupt hydrogen bonding (e.g., in proteins) in intra- and intercellular compartments of the skin [116].

While physical and nanotechnological approaches like those addressed in previous sections undeniably lie at the forefront of dermal and transdermal drug delivery research, CPEs remain the simplest and most cost-effective way to permeate different solutes across the skin, and their use is widely disseminated [116]. This explains the strong interest towards a better understanding of their modes of action [10,117] and on development of novel CPEs, searching for greener and more biocompatible alternatives, such as those derived from essential oils [118] or amino acids [4].

The wide diversity and relevance of CPEs recently motivated Bozdaganyan and co-workers to create an Open Access CPEs database, CPE-DB (http://intbio.org/cpedb/, last accessed on 4 November 2021), that includes ca. 650 CPEs covering all classes shown in Table 2 and a few more miscellaneous structural types [119].

Amidst the chemically diverse CPEs known to date, examples are found that can be categorized as ionic liquids (ILs), where organic cations are paired with organic or inorganic anions [120]. One emblematic IL that is widely employed as a topical antiseptic with high percutaneous permeation is cetylpyridinium chloride (CPC) [11], but others have also shown high potential as CPEs for DTD applications, including ILs based on natural building blocks like choline geranate (CAGE) [15,121] or obtained by pairing the [ionizable] drug itself with a proper counterion as in, e.g., dodecyldimethylammonium ibuprofenate [13]. Undeniably, ILs are rising stars for a broad variety of applications. As recently advocated, “The time is now for ionic liquids” [122]; the next section emphasizes this is also true for DTD of [bio]pharmaceuticals.

## 3. Ionic Liquid-Based Approaches in Dermal and Transdermal Drug Delivery

### 3.1. A Bird’s Eye View on Ionic Liquids

**Figure 9:**
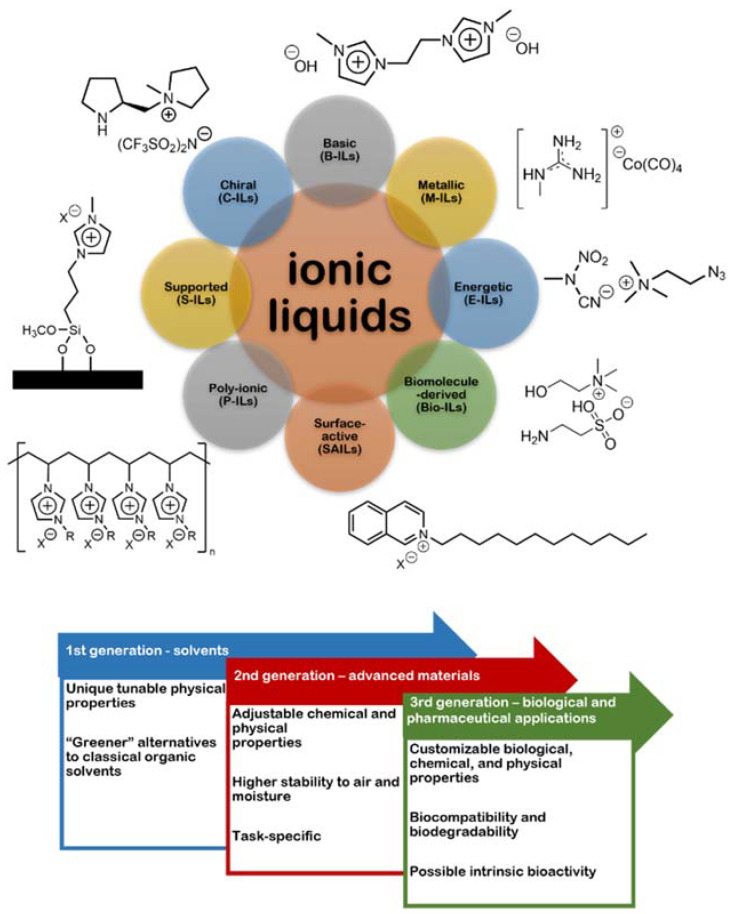
A few examples of different types of ILs, and their three recognized generations.

ILs are salts formed by organic cations and organic or inorganic anions, which possess unique physical and chemical properties that differentiate them from the other (“conventional”) salts. The most emblematic feature of ILs is their very low melting points (usually, but not necessarily, below 100 °C), due to a lack of ion symmetry and to low charge density, which results in Coulombic interactions in the solid phase that are weaker than those in other salts [14]. Given the huge number of possible cation/anion combinations, reports to date cover an immense panoply of different ILs that can be classified in many different ways, depending on the classification criteria (Figure 9), as thoroughly reviewed elsewhere [123]. In general, three generations of ILs have been recognized, going from the earlier examples of ILs proposed as “greener” surrogates of classical organic solvents to the next generation of ILs whose chemical and physical properties were adjustable to their specific applications (task-specific ILs, or TSILs), and finally to ILs displaying low toxicity, biocompatibility, biodegradability and, in some cases, even bioactivity [120,121,123,124,125].

ILs are particularly appealing for pharmaceutical applications, given their (i) non-crystalline nature, avoiding fluidity/polymorphism-related liabilities of many drug formulations, and (ii) easy customization and adjustable-properties, through a sensible choice of the ion components [12,124,125]. Hence, ILs properties such as vapor pressure, thermal stability, chemical and electrochemical stability, polarity, solubility, amphipathicity, and even bioactivity, can be finely tuned [14]. For instance, ionizable active pharmaceutic ingredients (APIs) have been paired with selected counterions to deliver ILs, including room-temperature ILs (RTILs), that possess intrinsic bioactivity [13,125,126]. Also, amphipathic ILs have been developed that are miscible in a wide range of solvents and display surface activity (surface-active ILs, or SAILs), representing a new class of surfactants, some of which possess interesting biological properties, such as antimicrobial action. Relevantly, though emerging from the 2nd generation of ILs for applications as, e.g., emulsifiers, SAILs enclose a tremendous potential for biomedical and pharmaceutical applications [14,126,127].

### 3.2. Ionic Liquids in Skin Permeation—A Closer Look at the Past Couple of Years

Over the last decade, the interest on using ILs to promote topical delivery of bioactive molecules and APIs has been steadily growing. For instance, several reports from 2010 to 2018 highlight the promising role of ILs, most of which are imidazolium-based, towards enhanced percutaneous permeation of drugs and bioactive compounds as diverse as acyclovir, methotrexate, dantrolene sodium, etodolac, 5-fluoroacyl, salicylic acid, caffeine, dencichine, peptides and proteins. Interestingly, some of these reports address studies on skin permeation of API-ILs, i.e., ILs resulting from combination of an ionizable drug (or API) with an adequate counterion (e.g., lidocaine chloride), or even of two ionizable APIs with opposed polarities (e.g., lidocaine docusate, lidocaine ibuprofenate, or lidocaine/etodolac) [128,129].

The mechanisms through which ILs or API-ILs display enhanced skin permeation are not fully unveiled and are primarily—though not exclusively—dependent on the specific structural and physico-chemical features of the IL. Thus, the CPE action of hydrophilic ILs (e.g., 1-ethyl-3-methylimidazolium-based) has been mostly ascribed to their role as polar enhancers able to (i) increase solubility and partition of hydrophilic drugs, and/or (ii) fluidize the SC by disrupting the tight packing of both proteins and lipids (at the headgroups level—Figure 8). In turn, hydrophobic ILs, many of which are SAILs (e.g., CPC, 1-dodecyl-3-methylimidazolium-based ILs), may exert their skin permeation action by (i) increasing solubility and partition of hydrophobic drugs, (ii) inserting into the SC lipid bilayers, causing a disruption of the ordered packing of the phospholipid bilayers or even phase separation or induction of lamellar phases (Figure 8), and/or (iii) extracting SC lipids [124,128,129,130,131]. Interestingly, SC lipid extraction followed by replacement of the extracted lipids with the IL and water has been recently advanced as the mechanism by which CAGE enhances the skin permeation of macromolecules like dextran [132].

ILs alone, and in combination with other percutaneous permeation methods, have been lately explored for DTD of diverse [bio]pharmaceuticals, so a few illustrative examples reported in the past couple of years are next highlighted.

### 3.3. IL-Based Topical Delivery Approaches for Small Bioactive Compounds

**Figure 10:**
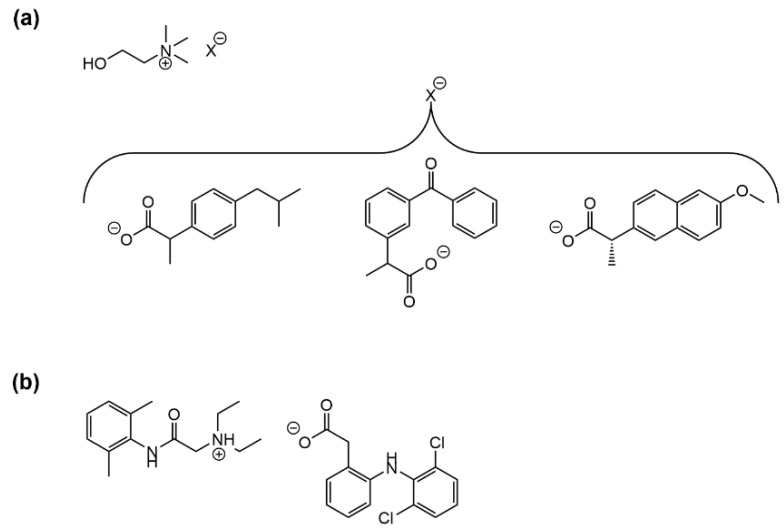
Examples of NSAIDs-derived ILs advanced for transdermal delivery: (**a**) ILs produced by pairing the cholinium cation with the anionic forms of (from left to right) ibuprofen, ketoprofen, and (*S*)-naproxen [135]. Adapted with permission from ref. [135]. Copyright 2019 American Chemical Society; (**b**) dual-action IL composed by the cationic form (conjugate acid) of lidocaine and the anionic form (conjugate base) of diclofenac [136]. Adapted with permission from ref. [136]. Copyright 2020 Springer Nature.

Non-steroidal anti-inflammatory drugs (NSAIDs), often ibuprofen but also others, are amongst the most common low molecular weight bioactive compounds that have been explored for IL-mediated topical delivery [125,130]. In this connection, Wu et al. have recently investigated the role of the counterion in the physico-chemical and biological properties of ibuprofen-derived ILs, with emphasis on transdermal delivery applications [133]. This study, which integrated nine different ibuprofen-based ILs using tetraalkylammonium and tetraalkylphosphonium counterions, confirmed the superior permeation of the ILs accross an in vitro skin model as compared to the free drug, highlighting the didecyldimethylammonium and tetrahexylammonium counterions as the most beneficial for such an effect [133]. Moreover, it was possible to establish a correlation between percutaneous permeation efficiency and (i) ILs‘ lipophilicity (higher logP and lower water solubility), and (ii) strength of ionic association between the anionic form of the drug and the cationic counterion [133]. In another latest study, Yuan et al. established that even minor amounts of choline/amino acid-based ILs, namely, cholinium glycinate and cholinium alaninate, contribute to a significant enhancement of the water solubility and permeation of ibuprofen across a model skin membrane, along with the advantage of showing low toxicity to mouse embrionyc fibroblasts [134]. Cholinium-based ILs have also been the focus of another quite interesting recent approach for enhanced DTD of NSAIDs, by Silvestre and co-workers [135]. These authors synthesized a series of API-ILs derived from ibuprofen, ketoprofen, and (*S*)-naproxen, using cholinium as the counterion, (Figure 10a) whose aqueous solubility were about one order of magnitude higher than those of the free NSAIDs. Moreover, incorporation of the API-ILs into bacterial nanocellulose afforded flexible and transparent membranes with adequate properties for use as DTD systems, while offering equal anti-inflammatory potency and a faster drug release as compared to loading of the parent NSAIDs alone [135].

NSAIDs other than the profens (2-aryl-propionic acid derivatives) have also been the subject of recent investigations addressing IL-mediated topical delivery. For instance, Carneiro and co-workers have reported the synthesis and characterization of diclofenac imidazolium monohydrate, an API-IL with increased water solubility as compared to the parent API that the authors advocate as promising for transdermal delivery approaches, although its cytotoxicity remains to be evaluated [137]. Current research is also addressing pairing diclofenac with local anesthetics or analgesics to afford dual-action ILs that are next embedded into suitable materials for topical application. For example, Suksaeree and Maneewattanapinyo have recently reported the ion-pair reaction between lidocaine hydrochloride and diclofenac sodium (Figure 10b) to afford a dual-action diclofenac/lidocaine IL that was next incorporated into a polymer matrix suitable for fabrication of transdermal patches [136]. The authors investigated the influence of the polymer matrix composition onto IL loading capacity, controlled drug release rate, and drug crystallization, advancing a few guidelines for future development of similar formulations [136]. The same research group further tested the controlled release of the diclofenac/lidocaine IL from gelatin/poly(vinyl alcohol) transdermal patches, which conveyed high release rates for both APIs and suitable physicochemical and stability features for pharmaceutical applications [138].

Incorporation of lidocaine/NSAIDs-based dual-action ILs into polyvinylidene fluoride/hyaluronic acid-based membranes has also been addressed by Abednejad et al., who found (i) an up to 470-fold enhancement of the water solubility of the ILs as compared to the free APIs, (ii) improved release of the APIs from the IL-loaded membranes, (iii) suitability of the IL-loaded membranes for use as wound-dressings, (iv) enhanced membrane adhesion and viability of membrane-adhered fibroblasts, and (v) anti-inflammatory activity similar to that of the free APIs [139].

IL-based strategies have also been lately addressed for enhanced skin permeation of other types of small bioactive compounds, from anticancer or neuroactive drugs to antioxidant or other anti-ageing agents, among others. For instance, amino acid-based ILs have been recently considered for topical treatment of cancer. In this regard, Zheng et al. have recently screened 15 methyl amino acid ester hydrochlorides as potential CPEs for model drugs like 5-fluoroacyl, and found that the L-proline and the L-leucine-based ILs were the most promising of the set, owing their percutaneous permeation ability to a combined lipid fluidization and lipid extraction effect [140]. Another amino acid, taurine, has been recently investigated for production of ILs with intrinsic antitumor activity and enhanced percutaneous permeation [141]. To this end, taurine was paired with bioactive alkaloids, namely, L-carnitine and betaine, affording ILs with high thermal stability, biocompatibility, and in vitro antitumor activity. The taurine-derived ILs were further shown to enhance percutaneous permeation of both insulin and dextran, possibly via a lipid extraction mechanism [141].

IL-mediated topical delivery of neuroactive molecules such as the memory-enhancing agents donepezil and nobiletin has been equally investigated in the last couple of years. Wu et al. ion-paired donepezil with docusate, ibuprofen, and unsaturated fatty acids, producing ILs that were thoroughly characterized regarding their structural and physicochemical properties, as well as in vitro antiproliferative action on human neuroblastoma cells, acetylcholinesterase (AchE) inhibitory activity, and ability to permeate through model blood–brain and skin barriers [142]. Additional skin permeation assays were carried out using IL-loaded adhesive transdermal patches, and—taken together—results highlighted a higher skin permeability of donepezil α-linolenate and docosahexaenate as compared to free donepezil, which could be further enhanced by loading the ILs onto the adhesive patches [142]. Moreover, despite having slightly decreased anti-AchE activity, the ILs had similar or lower cytotoxicity than the free drug [142].

IL-promoted DTD of another memory-enhancing molecule, the poorly water-soluble flavonoid nobiletin, has been also tested recently by Hattori et al., using choline and geranic acid (CAGE); this IL established multiple hydrogen bonds with the drug, contributing to a substantial increase of its solubility [143]. Additionally, in vitro and in vivo assays confirmed the superior performance of CAGE, as compared to other CPEs, in enhancing the percutaneous permeation of nobiletin, whose bioavailability via the transdermal route was found to be 20-fold higher than oral administration of the crystalline form of the drug [143].

ILs have also been lately explored as potential CPEs of anti-ageing molecules, such as α-lipoic acid and other natural antioxidants. Fang and co-workers prepared different ILs by acid-base combination of α-lipoic acid with a series of amines, and the ILs subsequently formulated with a liquid oil mixture to form water-in-oil NEs [144]. Transdermal permeation of the NEs was assayed in vitro on whole skin, and on medical tape-stripped epidermal and dermal skin layers, and these assays, together with additional structural and rheological studies, showed that different IL-skin layers’ affinities accounted for distinct skin permeation and retention ability [144]. Still, globally, the IL-based NEs not only provided enhanced solubility and protection to this somewhat unstable drug, but also showed very good in vivo action against photo-induced skin ageing and collagen loss in rats [144].

Caparica et al. have equally advanced IL-based emulsions as promising tools for enhanced solubility and percutaneous permeation of natural antioxidants relevant in cosmetics, namely, ferulic acid, caffeic acid, *p*-coumaric acid, and rutin [145]. These authors explored eight different ILs, encompassing amino acids, choline, and imidazole building blocks, and, after setting their highest nontoxic concentrations, based on their in vitro toxicity to human keratinocytes, determined their concentration-dependent ability to solubilize the antioxidants, and the physicochemical properties of IL-loaded oil-in-water NEs. This showed that incorporation of the ILs in the NEs conveyed a higher drug load in all four cases, holding great promise for future exploration of these formulations, especially those based on imidazolium glycinate, for DTD of natural anti-ageing agents as those covered in the study [145].

**Figure 11:**
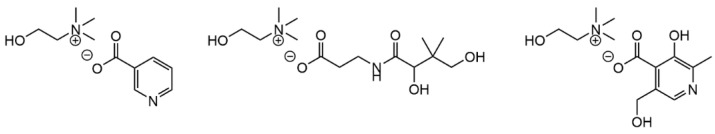
ILs combining the cholinium cation with the anionic forms (conjugate bases) of (from left to right) nicotinic acid (vitamin B3), pantothenic acid (vitamin B5), and pyridoxic acid (vitamin B6 co-factor), reported by Chantereau et al. [146]. Adapted with permission from ref. [146]. Copyright 2020 Elsevier.

Also with a focus on dermal care applications, Chantereau et al. reported, in early 2020, bioactive ILs obtained by pairing cholinium cations with anionic forms of B-complex vitamins, namely, nicotinate (vitamin B3), pantothenate (vitamin B5), and pyridoxilate (vitamin B6 co-factor) [146]. These vitamin B-derived ILs (Figure 11) showed significantly enhanced solubility as compared to the free vitamins, and their subsequent loading onto bacterial nanocellulose delivered flexible thermostable and nontoxic membranes with enhanced rehydration capacity as compared to non-IL-loaded bacterial cellulose. Moreover, in buffer, the release of the vitamin B-based ILs was faster and more extensive than that of the free vitamins [146].

One of the current greatest health concerns worldwide is that of MDR infections. In line with this, several latest reports address the use of IL-based strategies for topical application of diverse anti-infective agents to treat mild to complicated skin infections of viral, bacterial, or fungal origin. One example concerns acyclovir, whose clinical relevance in the management of herpes virus infections, including herpes labialis, is hampered by its very low water solubility. Hence, Islam et al. have recently reported the preparation of “ILs-in-oil” microemulsions (MEs) by combining hydrophilic choline-based ILs (“water”-like) with a mixture of the SAIL choline oleate and sorbitan laurate (“oil” phase), which allowed enhanced permeation of acyclovir through pig skin while showing no significant skin irritation [147]. The same authors have also investigated ternary systems comprising ethanol, isopropyl myristate, and choline/amino acid-based ILs, also with encouraging results for solubilization and skin permeation of acyclovir [148].

Many ILs have also intrinsic antimicrobial activity, which is putting them under the spotlight for the management of skin infections, including cSSTI. Imidazolium-based ILs have been for long known to possess antimicrobial action [12], although this has been occasionally hampered by their cytotoxicity [149]. Still, imidazolium-based ILs have been used to prepare novel formulations showing encouraging features to be advanced as safe transdermal delivery systems, e.g., IL-in-oil MEs based on 1-ethyl-3-methylimidazolium acetate [150], or intrinsic antibacterial and antibiofilm activity, as is the case of 1-butyl-3-methylimidazolium hexafluorophosphate-incorporated PLGA NPs developed by Takahashi and co-workers [151]. CAGE is another IL well-known for its activity as a CPE that was recently reported to display strong and broad spectrum antibacterial and antibiofilm activity, including against clinical isolates and MDR strains of bacterial species belonging to the so-called ESKAPE group (comprising *Enterococcus faecium*, *Staphylococcus aureus*, *Klebsiella pneumoniae*, *Acinetobacter baumanii*, *Pseudomonas aeruginosa*, and *Enterobacter* spp.) [152]. The antibiofilm action of CAGE was further investigated in vitro on biofilms of methicillin-sensitive *S. aureus*, revealing that the neat IL probably acts by contact killing, eradicating even 72 h-grown biofilms in less than a minute [152]. CAGE also shows promise for tackling skin fungal infections, as recently reported by Qi and co-workers [153]. These authors combined different antimicrobial ILs with the antifungal drug ketoconazole, and found CAGE as the most effective in both promoting deep skin penetration of the drug, and displaying a synergistic antifungal action in vitro against *Trichophyton interdigitale* [153].

Other IL-based approaches that are emerging in the latest literature are aimed at addressing topical administration of different types of anti-infective agents. For example, Zhang et al. have developed ME formulations based on the 1-hydroxyethyl-3-methylimidazolium chloride and lidocaine ibuprofenate ILs that were able to improve the transdermal permeation of the antimalarial drug artemisinin via disruption of the regular arrangement of keratin in the SC [154]. In another example, the 3-hexyl-1-vinylimidazolium bromide IL was employed in the production of a polymerized IL (P-IL) next used to fabricate MNs both possessing intrinsic antimicrobial activity and loaded with salicylic acid, for the topical treatment of skin infections associated to *Propionibacterium acnes*. These MNs were able to promote painless and prolonged DTD of salicylic acid, with enhanced anti-acne effects in both ex vivo and in vivo experiments [155].

Altogether, these examples highlight the tremendous chemical space that is to be explored regarding use of ILs for topical drug delivery, with focus on anti-infective approaches to face the rising menace of MDR pathogens. ILs are opening new avenues for the post-antibiotic era, as recently highlighted by Bento et al., [156] and one of those avenues may pass by IL-based approaches for topical delivery of antimicrobial and wound-healing peptides.

### 3.4. IL-Mediated Percutaneous Permeation of Biomacromolecules

One the general requisites for a molecule to be able to transpose the SC barrier to reach the viable epidermis and, eventually, deeper skin layers, is a maximum molecular weight of 1 kDa, which means that, unaided, [bio]macromolecules are unable to efficiently permeate across the skin [6]. This size limitation, along with solubility and stability issues, explains why many of the physical, nanotechnological, and chemical approaches addressed in Section 2 have been widely employed to enhance the percutaneous permeation of biomacromolecules such as nucleic acids, oligonucleotides, proteins, and peptides [6,157,158].

In connection with the above, ILs are also showing remarkable capabilities as new chemical tools for the DTD of polysaccharides, nucleic acids, proteins, and peptides. The potential of IL-mediated DTD of polysaccharides and proteins has been already hinted in the previous sub-section, when highlighting taurine-based ILs that, besides displaying intrinsic antitumoral activity, were also able to promote transdermal delivery of insulin and dextran [158]. Yet, polysaccharides like dextran, and related structures, seldom are the object of percutaneous permeation enhancement efforts. Still, Wu et al. have recently explored the potential of eight different choline-based ILs to mediate the DTD of a glycosaminoglycan, hyaluronic acid, to reduce skin dehydration [159]. The ILs were prepared via acid-base neutralization reactions using choline and selected natural acids (malic, sorbic, maleic, succinic, lactic, geranic, citric, and oleic), and cholinium citrate was found as the most capable of promoting penetration into deeper skin layers and, along with cholinium maleate, significantly reducing skin dehydration [159].

Choline-based ILs have been also lately explored for DTD of nucleic acids by Mitragotri and co-workers [160]. These authors prepared six ILs by a 1:2 cation/anion ratio mixing of cholinium bicarbonate, as the cation donor, with geranate, dimethylacrylate, isovalerate, isopropanoate, phosphate, or biphenyl-3-carboxylate anions, and next tested the ILs either individually or in different combinations, for their ability to promote DTD of a siRNA with therapeutic potential to tackle plaque psoriasis. The mixture combining CAGE and cholinium phenylpropanoate was the most efficient permeation enhancer that further showed high stability [160]. Additional in vitro and in vivo assays focused on this combination proved it as safe and able to efficiently silence the deviant gene, with observable decrease in psoriasis-related traits, such as thickened epidermis, inflammation, and hyperkeratosis, as compared to control mice [160]. Mitragotri’s group further investigated how ILs might contribute to the stabilization of framework nucleic acids (FNAs), whose emerging role as the next generation of precision-tailored and safe NCs for gene therapy of skin diseases is hampered by stability and percutaneous permeation limitations [161]. The authors combined the cholinium cation with the conjugate bases of six natural acids, namely, citronellic, glutaric, glycolic, octanoic, hex-2-enoic, and oct-2-enoic acids, via salt metathesis, and the six ILs thus obtained were tested in vitro and ex vivo for their ability to stabilize and permeate FNAs. Cholinium octanoate showed the most encouraging performance, being able both to keep the FNA NCs stable up to one week at room temperature, and to promote their delivery into the deeper layers of porcine skin [161].

Proteins and peptides are, by far, the most widely and deeply studied biomacromolecules, given their multiple pharmaceutical and biomedical applications, stemming from their broad range of structural, physicochemical, and biological properties, associated to a high level of specificity, thus representing the majority of biopharmaceuticals approved for therapeutic use [162]. Yet, most protein and peptides pose many challenges for therapeutic applications, mostly related to their low bioavailability and in vivo stability, which underpins intensive research on strategies for efficient protein and peptide delivery specifically aimed at oral and topical administration routes [158,163]. IL-mediated delivery of proteins has been investigated in recent years, with emphasis on insulin, since topical administration of this protein is highly convenient to promote higher comfort and compliance in diabetic patients. Favorable insulin permeation data were recently reported by Mitragotri and co-workers when using CAGE or taurine/carnitine ILs as, respectively, part of a biodegradable polymeric patch for transmucosal delivery [164], or percutaneous permeation enhancer [140]. Balcão and co-workers have also investigated the adequacy of CAGE and choline oleate ILs, prepared in a 1:2 cation/anion ratio, for potential use in transdermal delivery of insulin [165]. The ILs were evaluated regarding cytotoxicity, genotoxicity, oxidative stability, and ability to enhance insulin percutaneous permeation. CAGE presented the best profile, and was next incorporated in an optimized biopolymer formulation, affording a transdermal patch that efficiently promoted transdermal delivery of human insulin in a pig ear skin ex vivo model [165].

**Figure 12:**
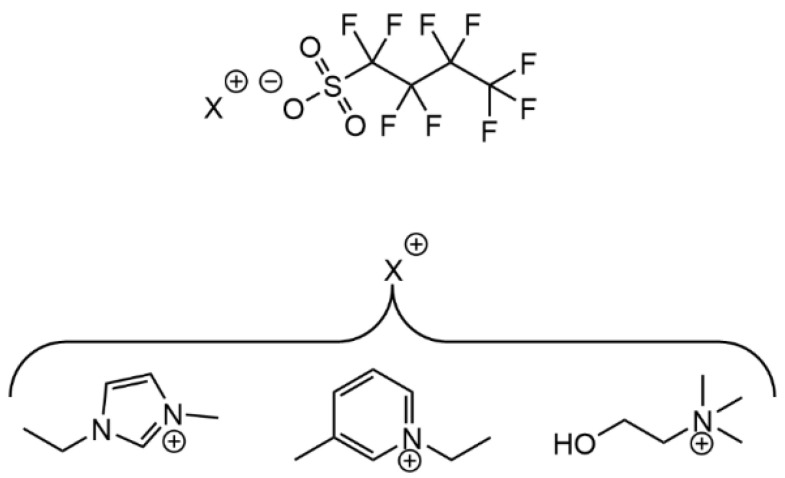
Perfluorobutanesulfonate-based ILs comprising the (from left to right) 1-methyl-3-ethylimidazolium, 1-methyl-3-ethylpyridinium, and cholinium cations, as reported by Vieira et al. [166]. Adapted with permission from ref. [166]. Copyright 2020 MDPI.

Another interesting report by Vieira et al. concerns the characterization of fluorinated ILs as potential protein DTD facilitators [166]. In this recent study, 1:1 combination of the perfluorobutanesulfonate anion with 1-ethyl-3-methylimidazolium, 1-ethyl-3-methylpyridinium, or cholinium cations (Figure 12) afforded ILs with high surface activity in aqueous media, i.e., SAILs. The self-assembling properties of these fluorinated SAILs were similar either in water or in buffered solutions of lysozyme, selected as the model cargo protein, which could be successfully encapsulated by the SAILs, except in the case of cholinium perfluorobutanesulfonate. Lysozyme release and activity studies showed the SAIL/protein systems to be reasonably stable for storage at 4 °C (no protein release up to 12 h with protein activity kept intact), whereas total protein release is observed after 12 h at 37 °C [166].

Like proteins, bioactive peptides hold great promise for therapeutic applications, with the further advantages of being more cost-effective and less prone to trigger immunoallergenic reactions. Interest is growing on topical delivery approaches for either antigenic peptides of interest for preventive vaccination, or host defense peptides (HDPs) encompassing antimicrobial and/or immunomodulatory effects [167]. Consequently, ILs are under the spotlight of the latest research on DTD of therapeutic peptides. For instance, Goto and co-workers developed hydrophobic fatty acid/amino ester-based ILs that were liquid at room temperature (i.e., RTILs) and fully miscible with common CPEs, such as isopropyl myristate (IPM) [168]. Formulations comprising 10% wt of the RTILs in IPM showed lower cytotoxicity than the standard CPE sodium lauryl sulfate and being also able to better permeate a NSAID (ibuprofen) than the conventional CPE Transcutol^®^. These formulations (especially the L-proline ethyl ester linoleate-based one), enhanced the percutaneous permeation of an antigenic peptide accross porcine skin [168]. The same authors have very recently used similar fatty acid-based ILs to formulate IL-in-oil nanodispersions that were optimized for higher physicochemical stability, as well as increased loading capacity and in vivo transdermal delivery of the anticancer nonapeptide leuprolide [169]. The nanodispersions showed no significant toxicity both in vitro and in vivo, and peptide transdermal delivery could be enhanced by as much as 65-fold compared with the aqueous delivery vehicle [169].

Tahara et al. have equally investigated fatty acid/choline-derived ILs as potential facilitators for the solubilization of an antigenic water-soluble peptide in an oil-based percutaneous permeation promoter [170]. The least cytotoxic IL cytotoxic, cholinium oleate, was dispersed with the peptide in the oil phase, and the resulting formulation showed a 28-fold enhancement of peptide transcutaneous permeation as compared to the aqueous vehicle. Moreover, this transdermal delivery formulation did not cause any detectable irritation on skin, and significantly suppressed tumor growth in vivo [170].

In regard to transdermal delivery of HDPs, latest efforts have focused on combination of antimicrobial HDPs with ILs possessing intrinsic antimicrobial activity, to afford new formulations whose percutaneous permeation and antimicrobial potency might be mutually enhanced. In this connection, Patel and co-workers mixed an HDP, melittin, with pyrrolidinium-based ILs, and the non-covalent HDP-IL conjugates thus formed displayed superior in vitro activity to those of their individual components against both Gram-negative (*E. coli*) and Gram-positive (*S. aureus*) bacteria, while showing no significant cytotoxicity [171]. The same authors further investigated the influence of the alkyl chain length of the pyrrolidinium-based ILs in both their individual antimicrobial potency, and synergistic action upon combination with melittin [172]. This study confirmed the potent synergic action against both *E. coli* and *S. aureus* when the HDP is combined with the ILs, and revealed a correlation between antimicrobial potency, which improved with the increase of the alkyl chain length in the IL [172]. Another example is the work reported by Gomes and co-workers [173]. In this study, coupling an antimicrobial methylimidazolium IL to the *N*-terminus of a collagenesis-inducing peptide with potent antibacterial and antibiofilm properties, delivered a covalent conjugate that retained the parent peptide’s activity against multidrug-resistant clinical isolates of Gram-negative bacteria, and antibiofilm action on a resistant clinical isolate of *Klebsiella pneumoniae*, while exhibiting much improved stability towards tyrosinase-mediated modifications [173]. These above-mentioned works are an overture for the potential held by IL-based strategies as tools to improve the properties of bioactive peptides.

## 4. Will Ionic Liquids and Peptides Become Relevant Co-Players in the Future Management of Skin Infections?

The encouraging findings highlighted in the last section, along with the latest reports on, e.g., (i) the fabrication of amphiphilic formulations comprising peptides and imidazolium- or betaninium-based ILs as new delivery nanoplatforms [174,175], and (ii) the relevance of developing cationic nanocarriers to overcome negatively-charged tissue barriers like the skin [176], hold great promise for the future management of skin infections, including cSSTI. Current progress in ILs-mediated percutaneous permeation of bioactive compounds, from small anti-inflammatory drugs to host defense peptides, and even lytic bacteriophages [177], together with the intrinsic antimicrobial action of many ILs, underline the capacity of ILs to become important players in innovative approaches to treat skin infections. Likewise, many HDPs combine antimicrobial, immunomodulatory and wound-healing properties, with a cationic amphiphilic structure often conveying cell-penetrating and/or self-assembling capacity. Hence, such HDPs will prospectively be useful both as carriers and as bioactive cargoes in transdermal delivery applications.

Effective management of infected wounds should ideally rely on topical formulations able to exert antimicrobial, anti-inflammatory, and healing effects from the outer to the innermost layers of the skin. ILs and HDPs have the potential to jointly contribute to such an achievement.

## Figures and Tables

**Table 1:** Examples of amphiphilic chemical permeation enhancers (CPEs).

Class	Examples	
	Name	Structure
Fatty acids	Lauric acid	CH_3_(CH_2_)_10_COOH
	Oleic acid	*cis*-CH_3_(CH_2_)_7_CH=CH(CH_2_)_7_COOH
	Linoleic acid	*cis,cis*-CH_3_(CH_2_)_4_CH=CHCH_2_CH=CH(CH_2_)_7_COOH
Fatty esters	Isopropyl myristate	CH_3_(CH_2_)_12_COOCH(CH_3_)_2_
	Isopropyl palmitate	CH_3_(CH_2_)_14_COOCH(CH_3_)_2_
Fatty alcohols	Octanol	CH_3_(CH_2_)_7_OH
	Decanol	CH_3_(CH_2_)_9_OH
Cationic surfactants	Cetylpyridinium chloride (hydrate)	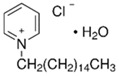
	Cetyltrimethylammonium chloride	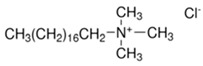
Anionic surfactants	Sodium lauryl sulfate	CH_3_(CH_2_)_11_OSO_3_^-^Na^+^
Zwitterionic surfactants	Lauryl betaine	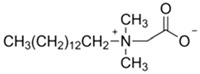
Non-ionic surfactants	Polysorbates (Tween^®^ surfactants)	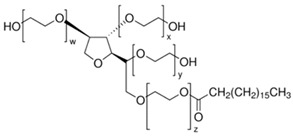

**Table 2:** Examples of different classes of CPEs and their recognized modes of action [116]. Adapted from the permission of ref. [116]. 2018 Haque and Talukder.

CPE Class	Example(s)	Mode(s) of Action
low molecular weight linear alcohols	ethanol hexanol octanol	extraction of intercellular lipids increased solubility/partitioning of the solute into the SC
glycols and polyols	propylene glycol polyethylene glycol	increased solubility/partitioning of the solute into the SC
esters	octyl salicylate	accumulation in the SC lipid bilayer enhancing solute diffusivity
amides	laurocapram (Azone^®^)	disruption of the ordered packing of the bilayers of skin lipids
sulfoxides	dimethylsulfoxide dodecyl methyl sulfoxide	extraction of intercellular lipids increased solubility/partitioning of the solute into the SC interaction with keratin and/or corneocytes, and with the polar head groups of the SC lipid bilayer
pyrrolidones	*N*-methyl-pyrrolidone 2-pyrrolidone	interaction with keratin and/or corneocytes, and with the polar head groups of the SC lipid bilayer
terpenes	menthol limonene nerol	disruption of the ordered packing of the bilayers of skin lipids
fatty acids	oleic acid	disruption of the ordered packing of the bilayers of skin lipids
fatty esters	isopropyl myristate propylene glycol monocaprylate propyleneglycolmonolaurate	extraction of intercellular lipids phase separation disruption of the ordered packing of the bilayers of skin lipids
surfactants	sodium lauryl or dodecyl sulfate (anionic)	interaction with keratin and/or corneocytes incorporation into the SC lipid bilayer and induction of lamellar phases
	quaternary ammonium salts (cationic)	significant disruption of the ordered packing of the bilayers of skin lipids
	cetyl or stearyl alcohol (nonionic)	disruption of the ordered packing of the bilayers of skin lipids
ether alcohols	2-(2-ethoxyethoxy)ethanol (Transcutol^®^)	insertion between the polar head groups of the skin lipid bilayers, inducing swelling of the SC
